# Alzheimer’s disease treatment: The share of herbal medicines

**DOI:** 10.22038/IJBMS.2020.50536.11512

**Published:** 2021-02

**Authors:** Masoud Soheili, Mohammad Karimian, Gholamali Hamidi, Mahmoud Salami

**Affiliations:** 1 Physiology Research Center, Institute for Basic Sciences, Kashan University of Medical Sciences, Kashan, Iran; 2 Department of Molecular and Cell Biology, Faculty of Basic Sciences, University of Mazandaran, Babolsar, Iran

**Keywords:** Alzheimer’s disease, Crocus sativus, Ginseng, Ginkgo biloba, Lavandula angustifolia, Magnolia officinalis, Melissa officinalis, Salvia miltiorrhiza

## Abstract

One of the most frequent forms of dementia in neurological disorders is Alzheimer’s disease (AD). It is a chronic neurodegenerative disease characterized by impaired learning and memory. Pathological symptoms as extracellular amyloid-beta (Aβ) plaques and intracellular accumulation of neurofibrillary tangles occur in AD. Due to the aging of the population and increased prevalence of AD, discovery of new therapeutic agents with the highest effectiveness and fewer side effect seems to be necessary. Numerous synthetic medicines such as tacrine, donepezil, galantamine, rivastigmine, memantine, glutathione, ascorbic acid, ubiquinone, ibuprofen, and ladostigil are routinely used for reduction of the symptoms and prevention of disease progression. Nowadays, herbal medicines have attracted popular attention for numerous beneficial effects with little side effects. *Lavandula angustifolia, Ginkgo biloba, Melissa officinalis, Crocus sativus, Ginseng, Salvia miltiorrhiza*, and *Magnolia officinalis *have been widely used for relief of symptoms of some neurological disorders. This paper reviews the therapeutic effects of phytomedicines with prominent effects against various factors implicated in the emergence and progression of AD.

## Introduction

Alzheimer’s disease (AD) is known as an epidemic problem throughout the world. It is one of the frequent forms of dementia disorder with progressive synaptic damage and neuronal degeneration (1, 2). There is a progressive increase in the prevalence of AD especially in ageing people such that about %4 of the populations have AD over the age of 65 and %47 after 85 years of age (3). The World Alzheimer Report in 2016 reported that about 47 million people are bearing physical, psychological, and behavioral problems related to AD, creating a heavy economic burden for both societies and healthcare providers (4). 

AD is characterized by learning and memory deficits, causing severe mental deterioration with social and occupational problems (5). The data acquisition and collection from the environment are known as learning, while programmed neural connections in the CNS leading to encoding, storing, and recovering information create memory (6, 7). Both are associated with cognition that is impaired in AD people. The interactions between age, education, genetics, and environmental factors affect AD (8). 

There are two main types of AD according to the age of occurrence; early-onset or familial form in the young and late-onset or sporadic form after 65 years. The early onset of AD, the autosomal dominant inherited form, is a rapid progression type of disease with shorter survival time compared to the sporadic form (9). Usually, the patients have a history of AD in their family and are frequently associated with genetic causes. Sporadic AD, as a highly polygenic disease, has slow progression with a prevalence rate of > 95% in all AD cases (10). It occurs due to extracellular accumulation of the 42 amino acid peptide, called amyloid-beta (Aβ) plaque (see below), related to APP cleavage imperfection or deficit in Aβ clearance. Moreover, ε4 isoform of Apolipoprotein E (Apo E4) is another significant genetic risk factor for sporadic form of AD. Apo E4 inhibits proteolytic degradation of Aβ through neprilysin (NEP) and insulin-degrading enzyme (IDE) activity (11). 

The main pathological features of AD are extracellular accumulation of Aβ plaques and intracellular formation of neurofibrillary tangles (NFT). In addition, glutamatergic and cholinergic dysfunction (12), oxidative stress (13), prion proteins (14), and inflammation (15) are implicated in the pathology of AD. 

Today, herbal medicine has attracted the attention of researchers due to its more effective therapeutic aspects along with fewer side effects compared with synthetic drugs (16-18). Multifunctional effects have introduced them as a therapeutic strategy for the treatment of a wide range of diseases. In this study, we have focused on some medicinal herbs known to be suitable for the treatment of AD.

**Etiology of AD**

***Amyloid-beta plaque***

Extracellular accumulation of Aβ plaque is one of the main histopathological hallmarks of AD. It is normally produced in the brain and has an important role in cell signaling and synaptic plasticity (19). Sequential enzymatic action on the transmembrane amyloid precursor protein (APP) by β- and γ-secretases causes Aβ generation (20). During aging, because of mutation in APP or related cleavage enzymes, including secretases and presenilin family, there is an overproduction of Aβ as well as a deficiency in its clearance, resulting in peptide aggregation and plaque formation (21). Excessive amount of Aβ fibrils induces neurotoxicity and synaptotoxicity, dysfunction, and degeneration of neurons and ultimately neuronal death (20). It also induces some abnormalities in brain metabolic processes leading to neuroinflammation (22). There are several different ways including microglial and macrophage phagocytosis, transcytosis across the blood-brain barrier (BBB), autophagy, and proteolytic degradation to clear Aβ from the brain (23). The ability of some degrading enzymes such as metalloendopeptidase, NEP, and IDE in the clearance of Aβ has also been demonstrated (24).

***Neurofibrillary tangles***

Neurofibrillary tangles are another fundamental neuropathological hallmark of AD. They are generated by hyperphosphorylation of the cytoskeletal microtubules associated protein called tau protein (25). Normally, tau proteins stabilize microtubules in neuronal pathways, however, in AD patients, hyperphosphorylation of tau proteins leads to formation of paired helical NFT filaments that stimulate host neuronal cell death (26). It is revealed that Aβ stimulates phosphorylation of tau and, therefore, provokes formation of NFT (27). Formation of NFT correlates with functional impairment, cognitive decline, and neurodegeneration especially in AD (28).

***Neurotransmission dysfunction***

***Glutamatergic system***

Glutamate is the most important excitatory neurotransmitter in the brain which is involved in different mechanisms of synaptic plasticity, the necessary process for encoding learning and memory phenomena (29). Dysfunction of the ionotropic N-methyl-D-aspartate (NMDA) glutamate receptor is importantly implicated in the neuronal excitotoxicity in AD (30). In late onset AD, Aβ can directly bind to the NMDA receptors, leading to increased extracellular glutamate concentration and excessive activation of the receptor (31). Overactivity of NMDA receptors itself disrupts Ca^2+^ influx leading to un-regulated intracellular signaling and neurotoxicity; a pathological mechanism recognized in some neurodegenerative disorders, including AD (32). It is also shown that Aβ oligomers have a toxic impact on glucose metabolism via AMP-activated kinase (AMPK). An impaired AMPK destroys synaptic plasticity through NMDA receptors which (33). In an Aβ independent manner, Apo E4 can occupy the NMDA receptors and impair synaptic plasticity in AD (34).

***Cholinergic system***

Acetylcholine (ACh) abundantly occurs in the brain synapses and is essential for brain processing and memory formation. It is synthesized and degraded by choline acetyltransferase and acetylcholinesterase enzymes, respectively (35). The level of ACh is shown to be declined in the cognition and memory relevant areas of the brain such as the cortex and hippocampus (36). Also, documents indicate that dysfunction of the cholinergic system is responsible for short-term memory deficit in AD (37). Importantly, in the latest stages of AD, due to decreased synthesis and increased degradation of ACh, the levels of the neurotransmitters decline by up to 85%. 

Cholinesterase enzymes that hydrolyze ACh exist in both neuronal and non-neuronal tissues; they are classified as acetylcholinesterase (AChE) and butyrylcholinesterase (BchE). Overactivity of AChE and BChE reduces ACh and disrupts the cholinergic system (38). Clinical evidence also shows that AChE can promote production and deposition of Aβ in AD patients (39). BchE, known as pseudocholinesterase, is a nonspecific cholinesterase enzyme involved in hydrolyzing of different types of choline esters. It is primarily associated with glial cells and endothelial cells in the brain with a minor role in regulation of brain ACh levels (40).

***Oxidative stress***

Oxidative stress is another mechanism through which the possibility of AD occurrence will be increased. Oxidative stressors cause damage to DNA, proteins, and other macromolecules (41). They cause mitochondrial dysfunction leading to excessive production of oxidative agents such as reactive oxygen species (ROS) and free radicals, leading to neurotoxic events (42) and autophagic degradation of mitochondria in AD people (43). Oxidative stress stimulates lipid peroxidation, a process that leads to formation of some reactive aldehydes like malondialdehyde (MDA) and 4-hydroxynonenal. They are known as major bioactive markers of lipid peroxidation and act as ROS. It is proven that the lipid peroxidation by-products play an important role in AD pathogenesis (44, 45). Plenty of studies have shown that many of the pathological symptoms of AD occur due to oxidative stress, which promotes the initiation and progression of AD (46-48). Indeed, a category of the proposed drugs treating AD are anti-oxidants which scavenge free radicals and prevent cells from damage (49). 

***Prion proteins***

Another effective mechanism involved in AD is prion (PrPc). This cell surface glycoprotein is found in neurons especially in the spinal cord (50). The normal function of PrPc is enigmatic but it seems that the PrPc acts as an anti-apoptotic and anti-oxidant protein (51). The expression of PrPc is regulated by the amyloid intracellular domain. PrPc reduces beta-secretase (BACE1) activity and prevents overproduction of the Aβ peptide. In AD patients, this interaction is disrupted, resulting in overactivity of BACE1, which in turn, leads to extra production of Aβ peptide (52). On the other hand, by binding to the ends of growing polymers, PrPc has an inhibitory effect on fibril elongation of Aβ. Interaction of Aβ oligomers with mutated PrPc plays a destructive effect on synaptic transmission (53) and inhibits memory consolidation (54). 

***Neuroinflammation***

Immunopathological investigations have proven that inflammatory mediators are increased in AD. High levels of complement proteins and acute phase reactants are detected in the brain of AD patients. Also transforming growth factor ß (TGF-ß), an anti-inflammatory cytokine that also regulates brain inflammatory mediators, is up-regulated in AD (55). 

Cyclooxygenase (COX) is a key enzyme responsible for brain inflammation in AD patients. It is shown that non-steroidal anti-inflammatory (NSAIDs) drugs play their anti-inflammatory role by inhibition of COX ll. Also, in AD patients, the amount of C-reactive protein and IL-6, as inflammation biomarkers, is considerably higher than in normal people, particularly in the early stages of the disease (56). 

Another important progressive biomarker of AD is the serum amyloid P component (SAP) which is produced in the liver and localized in the brain. SAP is a neurotoxic agent that, through binding to fibrils, protects Aβ from proteolysis. With disease progression, more cytokines and acute-phase proteins are released and, thus, more Aβ fibrils will be deposited (57, 58). 

**Current therapeutic methods**

Obviously, there are no absolute medications to reverse neuronal and synaptic destruction in AD (59), and currently approved drugs only alleviate clinical symptoms. The routine drugs for AD are cholinesterase inhibitors (60), NMDA receptor antagonists (61), and anti-oxidant and anti-inflammatory agents (62). These chemical synthetic drugs have various adverse effects such as nausea, diarrhea, bradycardia, and hepatotoxicity (63). Cholinesterase inhibitors such as tacrine, donepezil, galantamine, and rivastigmine are able to inhibit AChE, increase ACh concentration, and improve cognitive function (64, 65). Memantine, as a glutamate receptor antagonist, reduces Aβ deposition and disaggregates Aβ fibrils and thus, prevents neurotoxic effects. It also reduces neuronal cell death via Ca^2+^ influx regulation (66). Anti-oxidant agents such as glutathione, ascorbic acid, and ubiquinone scavenge free radicals and chelate metal ions, as well as preventing cell damage by ROS neutralization (67, 68). NSAIDs such as ibuprofen have a protective effect against the incidence of AD (69). Also, ladostigil is a chemical neuroprotective, anti-inflammatory, and neurogenesis inducing agent that is able to slow down the progression of mild cognitive impairment to AD (70, 71). Interestingly, in addition to synthetic drugs, current knowledge recommends the use of micronutrients (69, 70), supplements (73-71), and herbal medicines to relieve AD symptoms.

**Traditional phytomedicine**

In parallel to increasing concerns about the side effects of synthetic drugs, the tendency to use herbal medicines is growing. Although popularity of traditional treatment varies in different countries, the therapeutic role of herbs is under consideration worldwide (16, 18, 72). It is for a long time that herbal medicines have been used for the relief of brain disorders including AD (17, 73). In this context, albeit scant, attempts have been made to examine if herbal medicines have a considerable role in the treatment of AD. Here, we review recent findings from animal and clinical research about protective and therapeutic effects of several herbal medicines on AD. Behavioral, biochemical, cellular, and molecular aspects of investigations are considered. Henceforth, we evaluate the characteristics of some available and routinely used herbal medicines and their role in the treatment of AD.

***Lavandula angustifolia Mill.***

**Figure 1:**
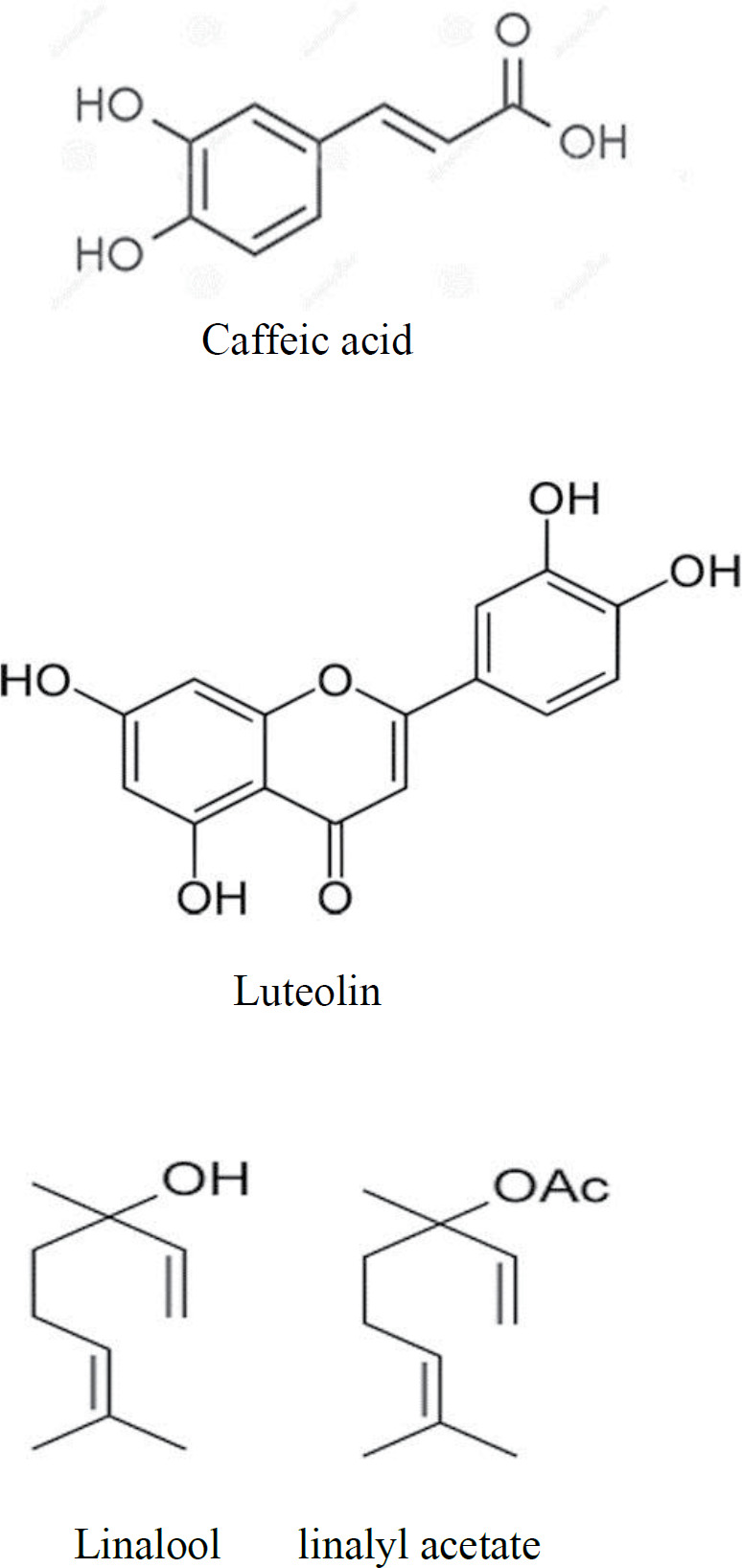
Chemical structure of caffeic acid, luteolin, linalool, and linalyl acetate (77, 85)

*Lavandula angustifolia* (lavender ) is a native aromatic shrub in the Mediterranean region that belongs to the *Lamiacea*e family (74, 75). Different extract forms of this plant including essential oil, aqueous extract, alcoholic extract, hydroalcoholic extract, and phenolic extract have been used in traditional treatment. While the main constituents of the essential oil of lavender are linalool and linalyl acetate, aqueous extract of lavender primarily consists of caffeic acid and luteolin (Figure 1) (76, 77). Our study indicated that the aqueous extract of lavender has no toxic effect on the Hep G2 cell line (75).

Numerous characteristics of herbal drugs such as anti-inflammatory (78) and anti-oxidant activities (79), inhibition of glutamate-induced neurotoxicity (80), prevention of Aβ polymerization (73),  anti-oxidant properties (77), and AChE inhibitory effect (80) have encouraged researchers to focus on lavender as a candidate medicine for the treatment of AD. 

It is reported that treatment of rat pups’ cerebellar granular cell culture with aqueous extract of lavender diminishes glutamate-induced neurotoxicity (76). It is shown that, via scavenging free radicals, lavender aqueous extract displays a potent anti-oxidant effect (77). 

In the level of neuronal activity, administration of aqueous extract of lavender in the Aβ injected rats restored deteriorated plasticity of hippocampal glutamatergic synaptic transmission (1). Also, intracerebroventricular injection of Aβ altered hippocampal protein expression in the hippocampus (81). Importantly, in an *in vitro *study using an atomic force microscope, we found that aqueous extract of lavender dose-dependently inhibits polymerization of Aβ monomer and prevents thickening of the Aβ fibrils (77). Further, histological assessment proved that lavender aqueous extract substantially clears brain Aβ plaques in the rat model of AD (82). In our previous study, using a Morris water maze task, we showed that aqueous extract of lavender improves impaired spatial learning and memory in an animal model of AD (5). A clinical trial, reported that lavender significantly reduced physical non-aggressive behaviors in patients with the dementia disorder (83). Metabolomic analysis of serum collected from the AD model of rats, receiving aqueous extract of lavender, showed that the extract restores metabolic profile of AD treated animals to normal status (84). 

Despite behavioral, electrophysiological, and histological evaluations confirming the favorable effect of lavender on the treatment of AD, due to different constituents of oil based, alcoholic, and aqueous extracts of lavender, caution must be exercised in using the herbal medicine. For instance, while the aqueous extract of lavender inhibits polymerization of the Aβ monomer, its essential oil promotes the formation of Aβ fibrils (73). 

***Ginkgo biloba***

*Ginkgo biloba*, or ginkgo, is a large tree with an angular crown and long erratic branches. This well-known traditional Chinese therapeutic herb has multi-functional effects. It has been used for thousands of years in folk medicine to treat a wide range of diseases (86). Several clinical investigations on AD patients validated the improving effect of *G. biloba* on cognitive impairment and disease progression especially in the early stages (87-89). It is shown that *G. biloba* can normalize ACh receptors in the hippocampus and stimulate the neurotransmitter activity leading to improvement of learning and memory in AD (90). A study showed the strong AChE inhibitory activity of *G. biloba* (91). The *G. biloba* extract protects brain cells against toxicity related to Aβ plaques (92) and affects some Aβ-induced events including ROS generation and accumulation, mitochondrial dysfunction, and apoptosis (92-94). Herbal medicine also inhibits free cholesterol circulation and interferes with Aβ synthesis (95). Consistently, research found that extract of the plant prevents *in vitro* Aβ oligomerization and fibril formation (93). Some evidence indicates that *G. biloba* can inhibit Aβ production by stimulation of the gamma-secretase pathway in the APP cleavage process (96). Moreover, *G. biloba* can protect astrocytes of rat hippocampus (97) and displays a neuroprotective effect through regulation of tau phosphorylation, elimination of amyloid plaques (98), induction of growth factors synthesis, and calcium homeostasis (99). 

The free radical scavenging effect of *G. biloba* has been proven by numerous *in vitro* and *in vivo* studies (94, 100-103). The medicinal plant increases the activity of anti-oxidant enzymes such as superoxide dismutase (SOD) and catalase (101). 

Glutathione (GSH) is a critical anti-oxidant agent in humans, animals, plants, fungi, and some bacteria and archaea. It is produced by the reduction of glutathione disulfide to sulfhydryl form of GSH enhanced by glutathione reductase. GSH is capable of preventing oxidative stress damage to cellular components.  It is proven that *G. biloba* enhances the activity of glutathione reductase and stimulates generation of GSH (104, 105). Another important mechanism implicated in various neurodegenerative diseases is the apoptotic pathway. It is reported that, through maintenance of mitochondrial membrane integrity and inhibition of cytochrome c releasing, *G. biloba* appears as an anti-apoptotic agent (101, 102, 106).  It prevents formation of the pre-apoptotic complexes and related caspase cascade.

**Figure 2:**
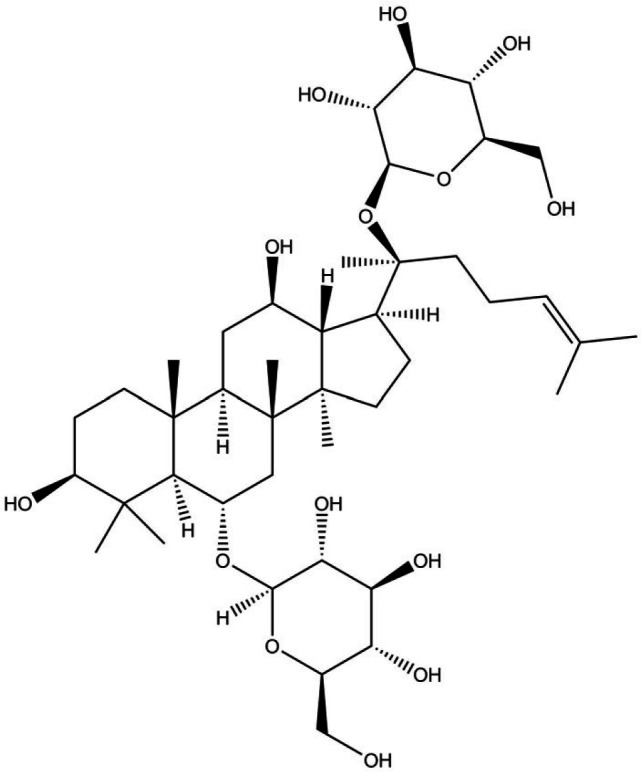
Chemical structure of ginkgolide (109)

Plentiful evidence indicated that ginkgolide (Figure 2) and flavonoids, as biological terpenic lactone components of this herb, display very specific and potent anti-inflammatory effects through antagonist activity on platelet-activating factor, a regulator of pro-inflammatory cytokines synthesis (107). 

Some other neuroprotective effects of *G. biloba* are protection against H_2_O_2_, NO, glutamate-induced toxicity, and hypoxia, as demonstrated in cultured neurons (108). 

***Melissa officinalis***

*Melissa officinalis*, also called lemon balm, belongs to the *Lamiaceae* family. This plant has small white flowers during summer and leaves with a mild lemon scent. This phytomedicine is used to ameliorate motivation and behavior in patients with dementia disorder (107). Anti-oxidant (110, 111), anti-depressant (112), anxiolytic (112), and anti-inflammatory (113) activities are attributed to this plant. The therapeutic effects of this herbal medicine are due to its main active constituents: triterpenes, phenolic acids, and flavonoids (114).

A Study by Lopez *et al.* confirmed that the aqueous and methanol extracts of *M. officinalis* diminish intracellular ROS generation (115). A study showed that some derivatives of *M. officinalis* such as flavonoids, caffeic acid, and rosmaric acid have anti-oxidant properties (116). It is demonstrated that medicine plays a potent anti-oxidant role through decreasing MDA (117), increasing GSH (118), and Paraoxonase 1, as critical enzymes in detoxifying oxidative stress mediators (117). It also displays anti-oxidant activity by scavenging free radicals, inhibiting lipid peroxidation, and protecting against H_2_O_2_ (119). 

It is reported that *M. officinalis* diminishes agitation and physical non-aggressive behavior in aged people (120) and modulates cognitive performance in healthy young volunteers (121). Soodi *et al.* demonstrated that the ethanol extract of *M. officinalis* enhances improvement of learning and memory in the scopolamine model of dementia. They attributed this cognitive supporting action to its inhibitory effect on AChE activity (122). *M. officinalis* alleviates neuronal excitability and improves cognitive dysfunction in AD patients (123). Research indicates that through affecting the serotonergic system and ligand-gated and ion channels, *M. officinalis* improves some symptoms in AD people (120, 124). Research proved that gallic acid, as an important constituent of *M. officinalis*, can reduce matrix metalloproteinase-2 activity that is involved in AD (125). The medicinal plant also shows a neuroprotective effect through reduction of Aβ induced neurotoxicity (126).

Taken together, different pharmaceutical effects of *M. officinalis* especially anti-cholinesterase, anti-oxidant, and anti-neurotoxicity activities have made herbal medicine an appropriate candidate for relieving symptoms of neurodegenerative diseases such as AD. 

***Crocus sativus***

**Figure 3:**
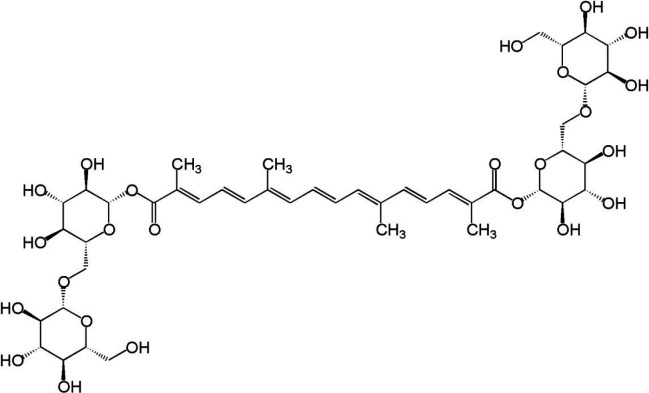
Chemical structure of crocin (131)

*Crocus sativus*, a species of *Iridaceae* family that is called saffron as well, has been widely used in traditional medicine. Anti-inflammatory (127), radical scavenging (128), and neuroprotective effects (129) are attributed to this herbal plant. The main sources of anti-oxidant activity of saffron are phenolic and carotenoids compounds (130). Most saffron effects, in fact, belong to one of the main active phytochemical ingredients called crocin (Figure 3) (131). Crocin plays multi pharmacological activities such as anti-oxidant (129), inhibition of peroxidized lipids formation (132), SOD activity restoration (133), neuronal protection (134), and neuron morphology preservation (135). This low stability compound is able to remove ROS powerfully (136). Experimental evidence proves the positive effect of crocin on memory and cognition improvement (137, 138), as well as plasticity of synaptic transmission in the neural circuits (139), a neural mechanism involved in learning and memory phenomena. This stemless flowering plant is shown to be effective in the treatment of mild to moderate depression (140) and mental illnesses (139).

Via suppression of inflammatory cytokines, *C. sativus* demonstrates anti-inflammatory properties. The attenuating effect of saffron extract on the production and deposition of Aβ in the hippocampus has been verified (141). It enhances up-regulation of lipoprotein receptor-related protein 1 and NEP enzymes. It can stimulate Aβ clearance by decreasing the tightness of BBB. It is found that, through anti-amyloidogenic activity (142) and anti-Aβ fibrilization (143), saffron plays an important role in the prevention of Aβ plaque formation in AD.

***Panax ginseng***

**Figure 4:**
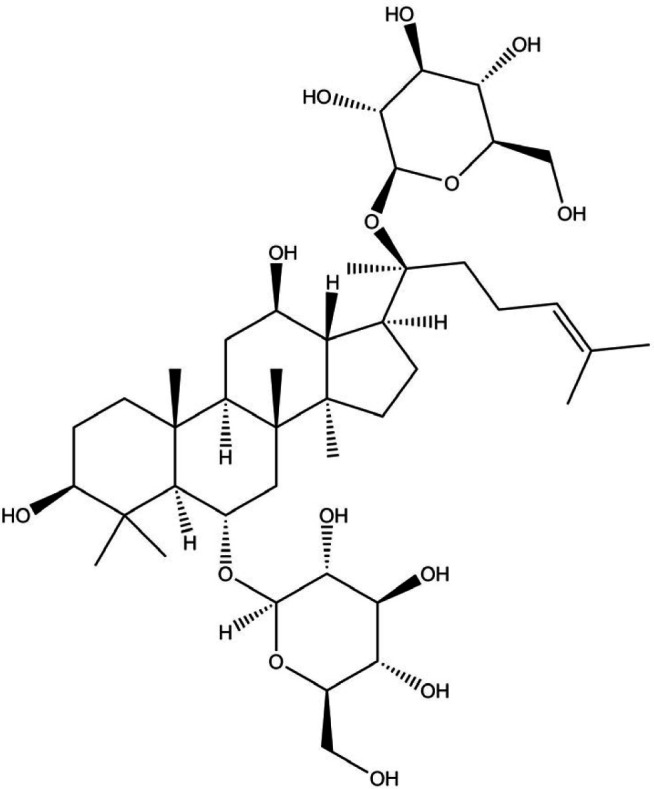
Chemical structure of ginsenosides (161)

*Panax Ginseng* is the root of a plant in the *Panax* genus that belongs to the *Araliaceae* family. It occupies a special place in ancient medicinal treatment (144). This hand-picked herb grows naturally in mountains and contains triterpene glycosides (Ginsenosides, Figure 4) responsible for main pharmacological activities (145). 

Aqueous extract of *ginseng* with polyphenol contents exhibits anti-oxidant activity (146). The herbal medicine scavenges free radicals such as superoxide anions and hydroxyl radicals and enhances the activity of the SOD enzyme (147). 

Choi *et al.* demonstrated that *ginseng* extract inhibits neuronal death and neuro-inflammation. With inhibition of hyperphosphorylation of tau protein, *ginseng* prevents formation of the neurofibrillary tangle. Further, it is able to inhibit the BACE1 enzyme and, therefore, reduce the level of Aβ (148). Ginsenosides increase hippocampal expression of brain-derived nerve factor (BDNF) (149), a key neuromodulator in learning and memory processing. Consistently, biochemical and behavioral evaluations have demonstrated that *ginseng* can improve stress-induced learning and memory impairment (150-152). Hence, it is proposed that the phytomedicine can play a distinct positive role in attenuation of memory impairment in AD (148).  

It has been reported that ginsenoside inhibits activity of AChE and BchE in cultured PC12 cell line resulting in increased amount of ACh content (153). In parallel, it restores choline acetyltransferase activity in an animal model of AD (154). It also shows neuroprotective ability where it suppresses the glutamate-induced toxicity in AD (155, 156). 

*In vivo and in vitro* studies have indicated that *ginseng* has anti-inflammatory activity through attenuating expression of inflammatory mediators such as TNFα, NF-κB, IL1β, and IL6 (157-159). It also decreases the level of the COX-2 enzyme, a key mediator in the inflammatory process (160).  

***Salvia miltiorrhiza***

*Salvia miltiorrhiza* is another member of *the Lamiaceae* family with branching stems and widely spaced leaves. It has been broadly used for treatment of various diseases (162-164). 

**Figure 5:**
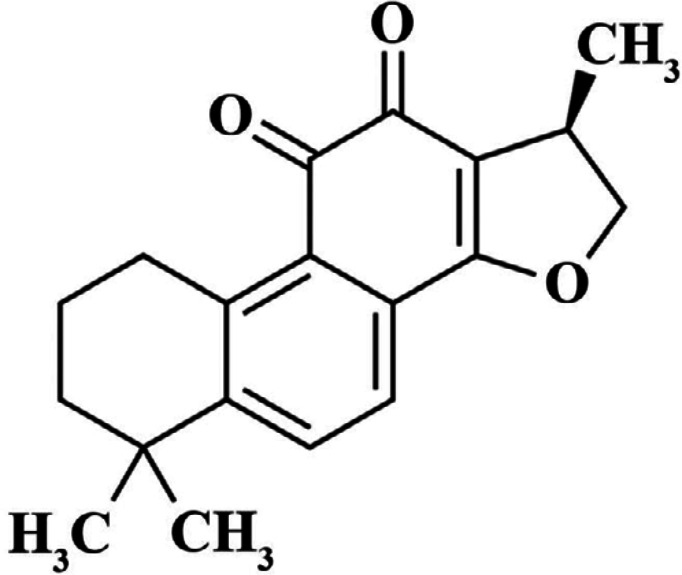
Chemical structure of Cryptotanshinone (185)

Cryptotanshinone (Figure 5), as the main active ingredient of *S. miltiorrhiza*, possesses many pharmaceutical functions including anti-AChE (165), anti-neurotoxicity (166), anti-inflammatory (167), anti-oxidative (168), and anti-apoptotic activities (169). Cryptotanshinone is reported to reduce Aβ deposition and improve spatial learning impairment (170). By affecting the gamma-secretase pathway, it prevents Aβ plaque formation and inhibits glutamate-induced neuronal toxicity (166). It is reported that cryptotanshinone ameliorates cognitive disturbance and significantly affect amnesia (165). 

Salvianolic acid is another polyphenolic derivative of *S. miltiorrhiza* that displays anti-inflammatory and anti-oxidant activity (171) and influences AD symptoms (172, 173). It dose-dependently prevents self-aggregation of Aβ and further disaggregates Aβ fibrils and protects cells against Aβ fibrils neurotoxic effect (174). Zhang *et al.* demonstrated that salvianolic acid increases BDNF expression and stimulates neuronal differentiation (175). Salvianolic acid protects the PC12 cell line against neurotoxicity induced by H_2_O_2_ and reduces lipid peroxidation and perseveres anti-oxidant enzymes, intracellular ca2+ level, and caspase-3 enzyme in the normal activity state (176). Zhang *et al.* demonstrated that salvianolic acid decreases leakage of lactate dehydrogenase and, hence, protects neuronal cells against H_2_O_2_ damage (177). 

Another constituent of *S. miltiorrhiza*, tanshinone, displays anti-oxidant activity. It can chelate metal ions that stimulate Aβ plaque formation and also inhibits ROS formation (178, 179). Tanshinone suppresses expression of inducible nitric oxide synthase (iNOS) and NO, and inhibits expression of inflammatory mediators (167, 180).  

It is reported that tanshinone has a strong preventive activity on the AChE enzyme (165) and significantly improves the amnesic activity in behavioral examination (181). Moreover, a study showed that tanshinone restores learning and memory deficit induced by scopolamine (182). Anti-apoptotic activity of tanshinone is shown to be due to down-regulation of caspase-3 expression or up-regulation of Bcl-2 expression (183). Tanshinone can activate the Bcl-xL pathway and, thus, through that, suppresses Aβ induced apoptosis (184). 

***Magnolia officinalis***

**Figure 6:**
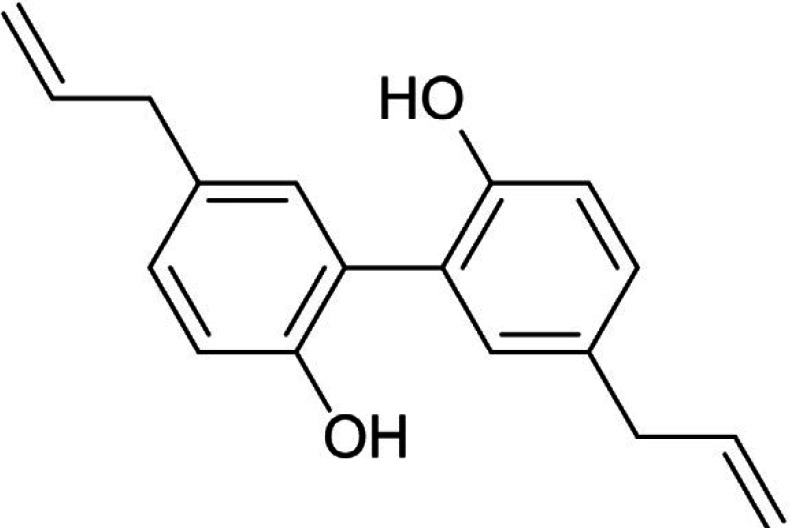
Chemical structure of magnolol (202)

*Magnolia officinalis* is a deciduous tree with thick and brown aromatic bark and fragrant flowers used as a rich source of biologically active compounds (186). This curative herb displays some medicinal aspects including anti-inflammatory (187), anti-oxidative (188), and neuroprotective activities (189). *M. officinalis* reduces the expression of inflammatory agents especially those stimulating NOS and inhibits activation of astrocytes and microglia (190). The multifunctional activity of *M. officinalis* is due to some ingredients such as magnolol (Figure 6), 4-O-methylhonokiol, honokiol, obovatol, and magnolol (191-193). It is reported that magnolia, as a major bioactive component of *M. officinalis*, positively impacts different oxidative agents and inflammatory cytokines, including ROS, iNOS, NF-κB, TNF-α, TGF-β, IL-1β, COX2, and MAP kinases family (191). It also up-regulates some proteins effective in anti-inflammatory activities such as Ras and Raf proteins (194). Honokiol, magnolol and 4-O-methylhonokiol display some neuroprotective effects through prevention of Aβ induced cell death, reduction of ROS generation, suppression of intracellular calcium elevation, and inhibition of caspase-3 activity (190, 195).

It is shown that the ethanol extract of *M. officinalis* has a preventive effect on Aβ accumulation in the mouse brain (196). It also inhibits the expression of BACE1 and therefore prevents Aβ production and has a hampering effect on memorial perturbation induced by Aβ plaque (196, 197). It was shown that 4-O-methylhonokiol prevents apoptosis induced by Aβ, resulting in cell survival, and down-regulates β-secretase expression and, thus, prevents Aβ formation. It also inhibits ROS generation and plays inhibitory action on H_2_O_2_ induced neurotoxicity (198, 199). Magnolol and honokiol inhibit AChE activity and stimulate release of ACh in the brain particularly in the hippocampus (200, 201).

It is documented that the ethanol extract of *M. officinalis* prevents memory deficit in an animal model of AD (190). Evidence indicates that ethanol extract of this medicinal herb reduces the level and activity of AChE in the cortex and hippocampus of mice treated with scopolamine (192). Table 1 summarizes the biochemical, histopathological, and behavioral effects of the herbal medicines in *in vivo* and *in vitro* studies.

## Conclusion

Taken together, extracellular Aβ plaque formation and intracellular accumulation of NFT are the main pathological features of AD. These structural abnormalities lead finally to neuronal death and synaptic loss which, in turn, result in violent neurobehavioral damages, mainly recent memory impairments. Oxidative agents, inflammatory factors, glutamate, or Aβ induced neurotoxicity, and cholinergic transmission deficit also promote occurrence of the diseases. Anti-oxidative, anti-inflammatory, and anti-neurotoxicity properties, as well as Aβ formation inhibitory and cholinergic excitatory activities of the herbal medicines are promising for the prevention and treatment of AD. As reviewed in this paper numerous herbal plants have potential therapeutic effects on AD associated symptoms. Despite abundant preclinical studies on the effectiveness of medicinal plants for neurodegenerative diseases including AD, clinical research is also required to warrant the use of herbal medicine in alleviating AD symptoms.  
